# Catheter ablation in combined procedures is associated with residual leaks

**DOI:** 10.3389/fcvm.2022.1091049

**Published:** 2023-02-01

**Authors:** Xuefeng Zhu, Wenjing Li, Hongxia Chu, Lin Zhong, Chunxiao Wang, Jianping Li, Pingping Liang, Lihong Wang, Lei Shi

**Affiliations:** ^1^Department of Cardiology, Yantai Yuhuangding Hospital, Yantai, Shandong, China; ^2^Doppler Ultrasonic Department, Yantai Yuhuangding Hospital, Yantai, Shandong, China

**Keywords:** atrial fibrillation, left atrial appendage closure, residual leak, catheter ablation, watchman

## Abstract

**Objectives:**

To compare patients with atrial fibrillation (AF) undergoing left atrial appendage closure (LAAC) with catheter ablation (CA) and those without CA.

**Background:**

The CA of AF may cause ridge edema, which may affect the safety of LAAC.

**Methods:**

Patients with AF (*N* = 98) who underwent LAAC (combined CA + LAAC procedure group; *N* = 51) or alone (LAAC group; *N* = 47) received pre-procedural, intra-procedural, and 6 week post-procedural transesophageal echocardiography (TEE). The depth and ostial diameter of LAA, device compression, residual leak, and ridge thickness were evaluated in the patients who had undergone combined and alone procedures, as well as images of LAA and primary clinical characteristics.

**Results:**

A residual leak was identified in 27 patients at 6 weeks after implantation by TEE (19 in the combined procedures group and eight in the alone group; *p* = 0.04). The combined procedure group had a significantly higher rate of a new residual leak than the alone group (25.5 vs. 8.5%; *p* = 0.03). Meanwhile, compared with at the time of implant, a smaller amount of device compression ratio was significant after 6 weeks (22.44 ± 3.90 vs. 19.59 ± 5.39; *p* = 0.03). There was no significant difference between both groups in all-cause mortality, cardiovascular mortality, and TIA/stroke/system embolism.

**Conclusion:**

The combined procedures of CA and LAAC for AF are feasible and safe; however, during the follow-up period, we found that the resolution of ridge edema caused by CA might cause an increased residual leak and a smaller device compression ratio.

## 1. Introduction

Patients with atrial fibrillation (AF) have a high risk of thromboembolic events and stroke (1) as well as a nearly 5-fold higher risk of stroke than those without AF (2). Management of AF crucially involves the prevention of stroke. In patients with non-valvular atrial fibrillation (NVAF), the origin of 90% of atrial thrombi seems to be from the left atrial appendage (LAA), as per autopsy and surgical data (3). To avoid thromboembolic events, every patient with AF at high thrombosis risk is required to take oral anticoagulants (OACs), although they exhibit disadvantages like acute hemorrhage and non-compliance (4). Therefore, technologies that can enhance the quality of life of patients with AF and prevent stroke need to be urgently developed. To prevent stroke, a potential alternative to OACs in patients with NVAF seems to be left atrial appendage closure (LAAC) because of the long-term efficiency and acceptable safety of the process (5–8). In patients with symptomatic AF, rhythm control can be efficiently achieved through catheter ablation (CA), although there are no reports of its role in the prevention of stroke (9), due to which continued long-term oral anticoagulation is recommended by clinical practice guidelines in these patients after CA therapy (10). Furthermore, a practical approach to the two left atrial (LA) interventions may be valuable because of general anesthesia, transseptal puncture, and the need for anticoagulants post-procedure (11). Recently, the safety and efficiency of a single procedure, the collateral therapy of LAAC for stroke prevention and CA for relief from AF symptoms, were assessed (12–14). However, the role of CA on the ostial size of LAA in association with the selection of device size and outcomes of the procedure, such as residual leaks, has not been discussed.

## 2. Materials and methods

### 2.1. Study population

In this retrospective, single-center study, 98 patients with symptomatic AF were enrolled between February 2016 and November 2019. All the patients were screened before, in between, and 6 weeks after surgery of the TEE procedure. Each patient who underwent LAAC was grouped either into the combined procedures group (*N* = 51) or the alone group (*N* = 47). Patients eligible for combination therapy were as follows: (1) patients with refractory NVAF and (2) patients with one of the following conditions: (a) CHA2DS2-VASc score ≥2, (b) HAS-BLED score ≥3, (c) contraindications to long-term OACs, and (d) refusal of OACs as antithrombotic therapy based on personal preference, among which CHA2DS2-VASc score ≥2 is a required item and the other three are optional (either one of three) for choosing LAAC. The data, including clinical and demographic characteristics, the rate of procedural success, adverse events or periprocedural complications, and follow-up for the long term, were analyzed. The study followed the Declaration of Helsinki principles and international rules for scientific studies with approval from the Institutional Review Board at Yantai Yuhuangding Hospital. Each patient was informed about the procedures, and they gave their written consent.

### 2.2. Pre-procedural protocols

The indexed LA volume, the left ventricular dimensions, the right atrial size, and ejection fraction of patients have been retrospectively obtained from the transthoracic echocardiographic (TTE) images as per the echocardiography report. Before the procedure, transesophageal echocardiography (TEE) was used to confirm the absence of a thrombus in the LAA or LA. Baseline measurements of the depth and thickness of the ridge and LAA ostial diameter were obtained at standard 0, 45, 90, and 135 TEE views of the omniplane as described in a previous study (15). Besides the lobe number, we also characterized the LAA shape through TEE evaluation as either windsock, chicken wing, cactus, cauliflower, or mixed, as described earlier (16, 17). Each patient was administered novel oral anticoagulants (NOACs) (15 mg qd rivaroxaban or 110 mg bid dabigatran) as a single-held dose or uninterrupted therapeutic warfarin [international normalized ratio (INR) 2–3].

### 2.3. CA of AF

Before the LAAC, ablations of the AF were undertaken. As an analgesic, fentanyl was given, and lidocaine was administered as local anesthesia in the left subclavian and groin regions. A three-dimensional CARTO3 mapping system from Biosense Webster (Johnson & Johnson) was used to guide pulmonary vein isolation (PVI) point–by–point through a ThermoCool Smart Touch Catheter (Biosense Webster; Johnson & Johnson) at specific power (35–40 W) and temperature (43°C). The endpoint of CA was a bidirectional conduction block between the pulmonary vein (PV) and LA veins. In patients with paroxysmal AF, PVI along with non-PV trigger elimination was carried out. For patients with persistent AF, additional linear and/or complex fractionated atrial electrogram ablations were carried out. The sinus rhythm restoration was attained by ibutilide fumarate injection, ablation, or electric cardioversion.

### 2.4. LAAC procedure

**Figure 1:**
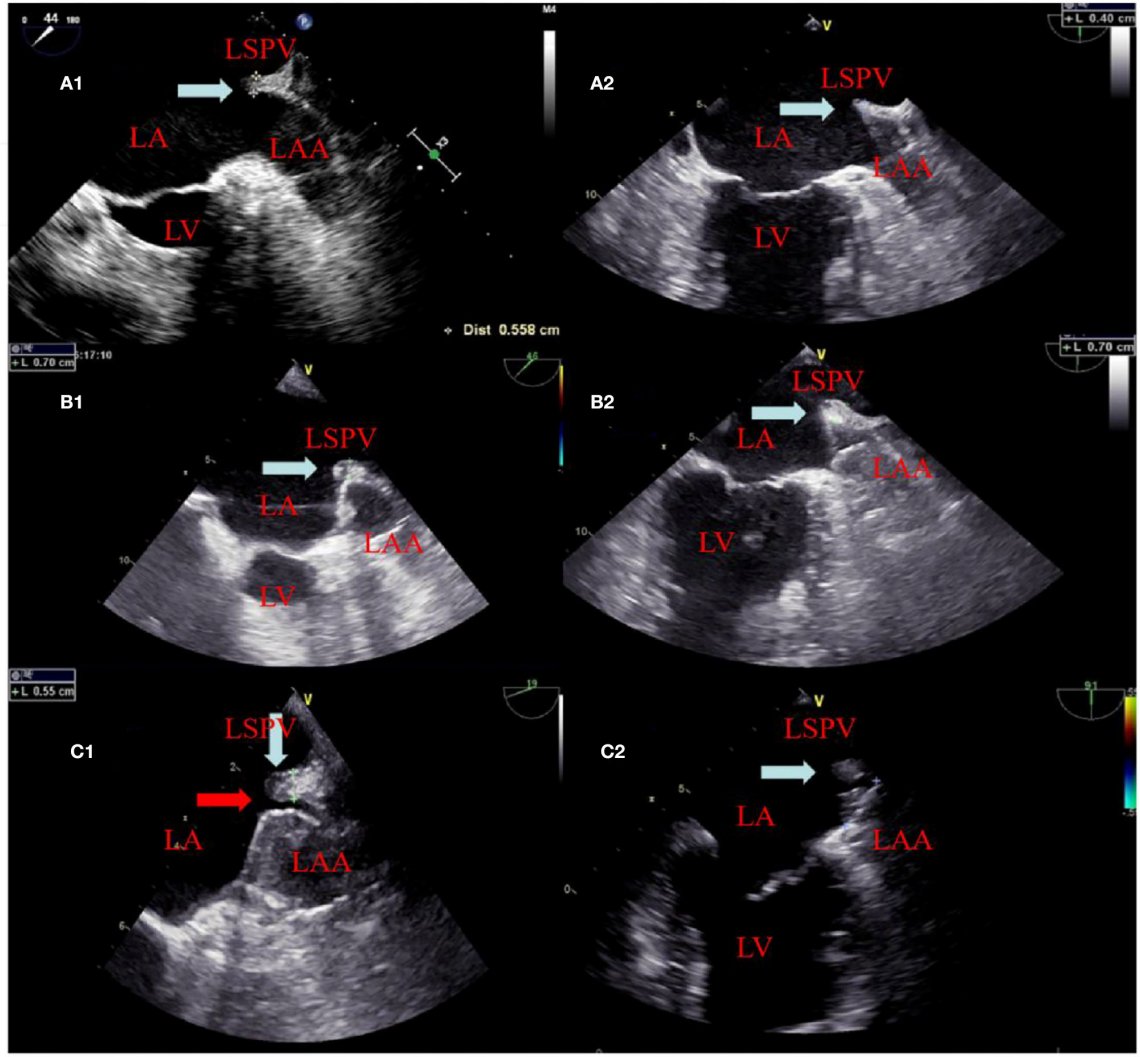
The change of the ridge and residual leak at different times. A panel of images showing appearances of the ridge (white arrow) between the left atrial appendage and left superior pulmonary vein before ablation in **(A1, A2)** and edema (white arrow) after ablation in **(B1, B2)**. **(B1, B2)** showed that the position of the device after deployment is close to and far from the ridge edema region, respectively. In **(C1)**, TEE showed ridge edema disappears and a residual leak of 2 mm (red arrow) at the 6 week follow-up. **(C2)** TEE also showed ridge edema disappears but did not affect the residual leak because it was far from the ridge edema region at the 6 week follow -up.

Left atrial appendage closure was performed after CA in the combined procedure group, while for the alone group, LAAC was performed directly. LAAC was operated by implanting WATCHMAN (WM) FLX legacy 2.5 from Boston Scientific (Marlborough, USA) as the plan under local anesthesia based on the information obtained by angiography and TEE. A 14-F WM delivery sheath was introduced into the LAA, followed by the advancement of a catheter (5-F pigtail) into the most distal portion of the LAA to carry out an LAA angiography at a caudal angle of 20–30° and a right anterior oblique angle of 20–30°, outlining the LAA size and shape. If the device orientation showed that the occlusion was favorable, the same transseptal puncture was used; otherwise, a new transseptal puncture was performed. The size of the device was chosen to be 10–20% larger than the largest diameter of the LAA estimated through TEE guidance and angiography. The pigtail catheter was removed when the guide catheter tracked into the distal LAA over the pigtail. The retraction of the access sheath deployed the WM before the device was unsheathed with full deployment, ensuring proper positioning. The features of the device were then analyzed under the guidance of TEE and angiography; during the procedures, TEE was performed to confirm the LAAC. Residual leaks, device compression ratio, and thickness of the ridge were calculated immediately after the procedures (Figure 1). A residual leak of < 1 mm was designated as minimal, 1–2.9 mm as mild, 3–5 mm as moderate, and >5 mm as severe. The device was disconnected from the delivery catheter upon fulfilling suitable PASS criteria, including position, an appropriate seal on TEE and angiography, 10–20% compression, and stability on tug testing.

### 2.5. Follow-up of patients

After the procedure and 1 or 2 days of hospital stay, patients were discharged after excluding tamponade/significant pericardial effusion or major bleedings related to the procedure, and other acute periprocedural complications. After the procedure, OAC was recommended for 3 months in the combined procedures group and 6 weeks in the alone group. TEE was also performed 6 weeks after the procedure to measure residual leaks, device compression ratio, and thickness of the ridge in both groups. When the TEE exhibited no residual leaks (width of jet > 5 mm), absolute closure of the LAA, or no thrombus related to the device, the OAC then changed to the antiplatelet duplex therapy using clopidogrel (75 mg) and aspirin (100 mg) in both groups (after 3 months vs. after 6 weeks) until the sixth month, and then, to aspirin alone 6 months later. If a thrombus were detected, the regimen of anticoagulation was restarted with NOAC or warfarin and aspirin until the thrombus was fully resolved by regular TEE exams. Besides, each patient received an outpatient follow-up for the long term or was telephonically contacted to inquire about any clinical events, including TIA/stroke/events of systemic thromboembolism, bleeding, and death.

### 2.6. Statistical analysis

Collection and analysis of data on demography, procedures, echocardiography, and clinical follow-up were carried out. Data are expressed as a percent for categorical variables and counts and mean ± standard deviation were used to express continuous variables. Significant variation for continuous variables among the two groups was assessed through independent-samples Student's *t*-test; a comparison of the categorical variables was done through Fisher's exact test or a chi-squared analysis. SPSS version 23.0 from IBM Software, Inc. (NY, USA) was used for all analyses. A statistically significant value had a *p* < 0.05.

## 3. Results

### 3.1. Patient baseline characteristics

The characteristics of each patient who underwent LAAC were noted at baseline. As shown in Table 1, the HAS-BLED scores and mean CHA2DS2-VASc scores were comparable between groups (Table 1). Measurements of TEE of the maximal ostial diameter of LAA and depth for the procedure groups (combined and alone) at the omniplane 0, 45, 90, and 135 are listed in Table 2. Measurement of LAA through angiography of LAA maximal ostial diameter for the combined procedures group and the alone group are shown in Table 3. These results demonstrate no significant difference between the average LAA ostial diameter, the number of lobes, the depth of the lobes, LAA shape, and ridge thickness between the two groups. Similarly, there was no significant difference between the two groups in the pattern of AF of clinical comorbidities such as hypertension, chronic heart failure, diabetes, coronary heart diseases, previous stroke/TIA, and previous major bleeding.

**Table 1:** Baseline characteristics.

**Variable**	**Combined procedure group** **(*n* = 51)**	**Alone group** **(*n* = 47)**	**P-value**
Age, years (mean ± SD)	64.4 ± 9.44	64.9 ± 8.62	0.86
Female sex, *n*	26	24	1.0
BMI, mean ± SD, kg/m^2^	21.37 ± 1.87	21.24 ± 2.56	0.85
Chronic heart failure, *n*	12	9	0.63
Hypertension, *n*	31	25	0.54
Diabetes mellitus, *n*	14	9	0.35
Coronary heart disease, *n*	11	7	0.44
Previous TIA/stroke, *n*	37	35	1.0
Previous major bleeding, *n* (%)	3	1	0.62
CHA2DS2-VASc score (mean ± SD)	3.80 ± 0.95	3.65 0 ± 0.99	0.63
HAS-BLED score (mean ± SD)	3.5 0 ± 0.83	3.55 ± 0.89	0.86
Antiplatelet therapy, *n*	22	20	0.558
Antiarrhythmic drugs, *n*	37	31	0.313
Anticoagulation with warfarin, *n*	7	5	0.439
Anticoagulation with NOACs, *n*	23	18	0.390
LA size, mean ± SD, mm	41.15 ± 5.57	43.10 ± 2.81	0.17
LVEF, mean ± SD, %	57.30 ± 8.86	57.85 ± 6.34	0.823
Moderate-to-severe mitral regurgitation	8	5	0.332
Patent foramen ovale, *n*	7	5	0.439
Paroxysmal AF, *n*	16	18	0.53
Persistent AF, *n*	35	29	0.53
Abnormal renal function, *n*	3	2	0.539
Abnormal liver function, *n*	1	0	0.520
Rhythm of AF during LAAC	29	34	0.082

Values are mean ± SD or n (%). BMI, body mass index; TIA, transient ischemic attack; CHA2DS2-VASc, congestive heart failure, hypertension, age ≥ 75 years, diabetes mellitus, prior stroke or transient ischemic attack or thromboembolism, vascular disease, age 65–74 years, sex category; HAS-BLED, hypertension, abnormal renal or liver function, stroke, bleeding, labile international normalized ratio, elderly, drugs, or alcohol; LA, left atrial; LVEF, left ventricular ejection fraction; AF, atrial fibrillation.

**Table 2:** TEE measurements of LAA ostial diameter and depth and LAA morphology.

**LAA view**	**Combined procedure group** **(*n* = 51)**	**Alone group** **(*n* = 47)**	**P-value**
**Ostial diameter, mm**			
0	19.67 ± 2.38	19.92 ± 3.28	0.81
45	19.53 ± 3.54	19.23 ± 2.35	0.79
90	19.60 ± 2.92	18.92 ± 3.86	0.61
135	21.33 ± 3.64	21.54 ± 3.93	0.89
**Depth mm**			
0	26.56 ± 5.56	26.83 ± 5.36	0.90
45	26.71 ± 6.22	27.92 ± 5.68	0.61
90	28.36 ± 3.92	27.00 ± 5.29	0.46
135	25.41 ± 4.20	26.00 ± 5.95	0.67
**Number of LAA lobes**			
1	14	9	0.35
2	24	26	0.43
3	11	11	1.0
4	2	1	1.0
**LAA morphology**			
Cactus	9	7	0.79
Cauliflower	33	29	0.84
Chicken wing	8	10	0.60
Windsock	1	1	1.0

Values are mean ± SD or n (%). LAA, left atrial appendage.

**Table 3:** Procedural characteristics.

	**Combined procedure group** **(*n* = 511)**	**Alone group** **(*n* = 47)**	**P-value**
Success implantation (%)	51 (100)	47 (100)	1
Angiography LAA Ostial maximal diameter, mm	23.40 ± 4.05	24.53 ± 2.85	0.38
Device size	28.80 ± 4.06	30.00 ± 3.40	0.39
**LAA seal**			
Complete seal, *n* (%)	43 (84.3)	41 (87.2)	0.78
Residual leak minimal (< 1 mm), *n*	3	3	1.0
Residual leak mild (1–2.9 mm), *n*	3	2	1.0
Residual leak moderate (3–5 mm), *n*	2	1	1.0
Residual leak severe (>5 mm), *n*	0	0	1.0
Residual leak at 6 weeks, *n* (%)	19 (37.3)	8 (17)	0.04
Residual leak at 6 weeks minimal (< 1 mm), *n*	9	4	0.151
Residual leak at 6 weeks mild (1–2.9 mm), *n*	6	3	0.286
Residual leak moderate at 6 weeks (3–5 mm), *n*	3	1	0.340
Residual leak severe at 6 weeks (>5 mm), *n*	1	0	0.520
New residual leak at 6 weeks, *n* (%)	13 (25.5)	4 (8.5)	0.03
Persistent residual leak at 6 weeks, *n* (%)	6 (11.8)	4 (8.5)	0.74
Device compression at implant (%)	22.44 ± 3.90	22.85 ± 3.72	0.70
Device compression at 6 weeks (%)	19.59 ± 5.39	21.58 ± 4.37	0.15
Close to the ridge, *n* (%)	28 (54.9)	22 (46.8)	0.54
Pre-procedural thickness of ridge (mm)	4.91 ± 0.48	4.83 ± 0.56	0.67
Intra-procedural thickness of ridge (mm)	7.06 ± 3.90	4.83 ± 0.56	p < 0.01
Post-procedural thickness of ridge at 6 weeks (mm)	5.15 ± 0.41	4.83 ± 0.56	0.09
**Complications**			
Pericardial effusion, *n*	1	1	1.0
Hematoma, *n*	2	1	1.0
Device thrombus 6 weeks, *n*	1	0	1.0

LAA, left atrial appendage.

### 3.2. Results of the LAAC procedure

The procedural characteristics are listed in Table 3. The WM device was successfully implanted in all 98 patients. In the combined procedures group, ridge edema between the left pulmonary veins and the opening of the LAA was noted on TEE after ablation, as compared with the pre-procedure (Figure 1). The implantation of each device was successful, with no acute residual leak >5 mm, as detected through TEE. Acceptable residual leaks ( ≤ 5 mm) were detected in eight patients in the combined procedures group and six patients in the alone group at implantation (*p* = 0.78). The deployed device size in the combined procedures group and the alone group was 28.80 ± 4.06 vs. 30.00 ± 3.40 mm (*p* = 0.39), the pre-procedural thickness of the ridge was 4.91 ± 0.48 vs. 4.83 ± 0.56 mm (*p* = 0.67), and their compression ratio was 22.44 ± 3.90 vs. 22.85 ± 3.72% (*p* = 0.70), which was not significant between the groups. On the contrary, the intra-procedural thickness of the ridge was 7.06 ± 3.90 vs. 4.83 ± 0.56 mm (*p* < 0.01) in the combined procedure group, which was significant. The number of patients with the position of the WM device close to the ridge after deployment was 28 (54.9%) in the combined procedure group and 22 (46.8%) in the alone group (*p* = 0.54).

### 3.3. TEE follow-up results

**Figure 2:**
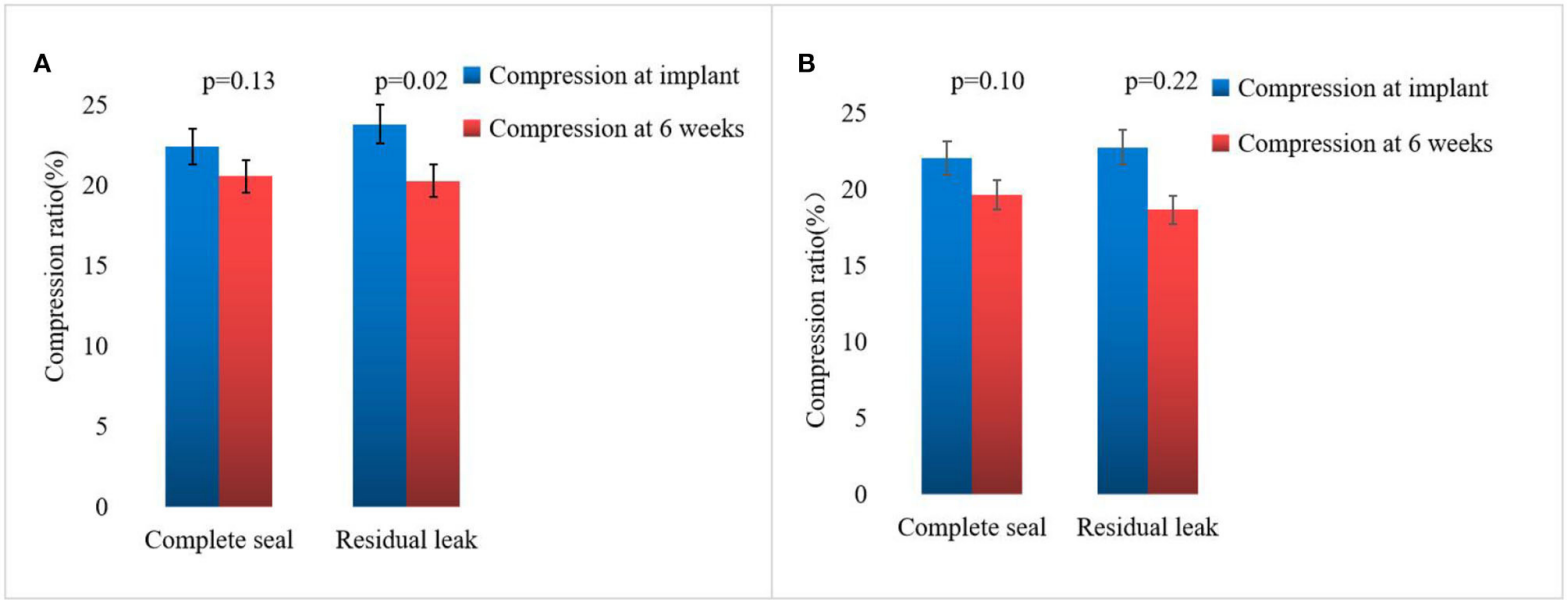
Compression ratio immediate and 6 weeks WM implant of complete seal vs. residual leak. **(A)** Comparison of immediate and 6 week follow-up WATCHMAN implant compression in the combined procedure group of complete seal vs. residual leak. **(B)** Comparison of immediate and 6 week follow-up WATCHMAN implant compression in the alone group of complete seal vs. residual leak. The bar graph represents mean ± SD.

**Figure 3:**
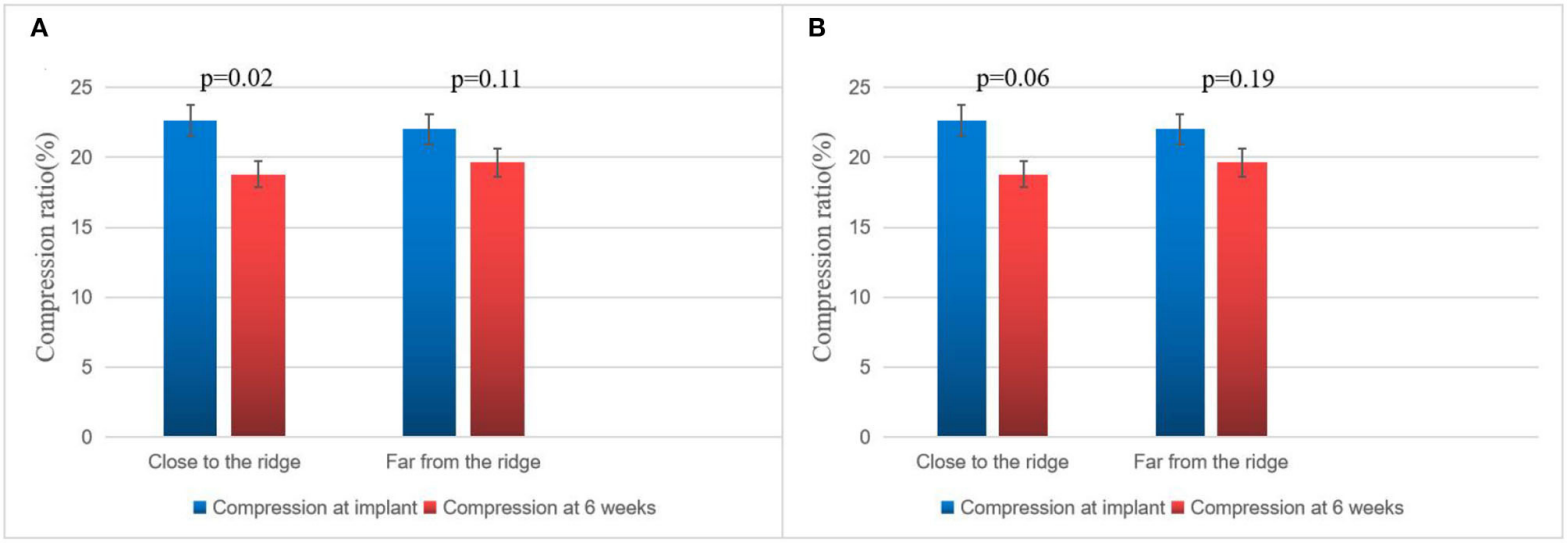
Compression ratio of immediate and 6 week WM implant close to the ridge vs. far from the ridge. **(A)** Comparison of immediate and 6 week follow-up Watchman implant compression in the combined procedure group of close to the ridge vs. far from the ridge. **(B)** Comparison of immediate and 6 week follow-up WATCHMAN implant compression in the alone group close to the ridge vs. far from the ridge. The bar graph represents mean ± SD.

**Figure 4:**
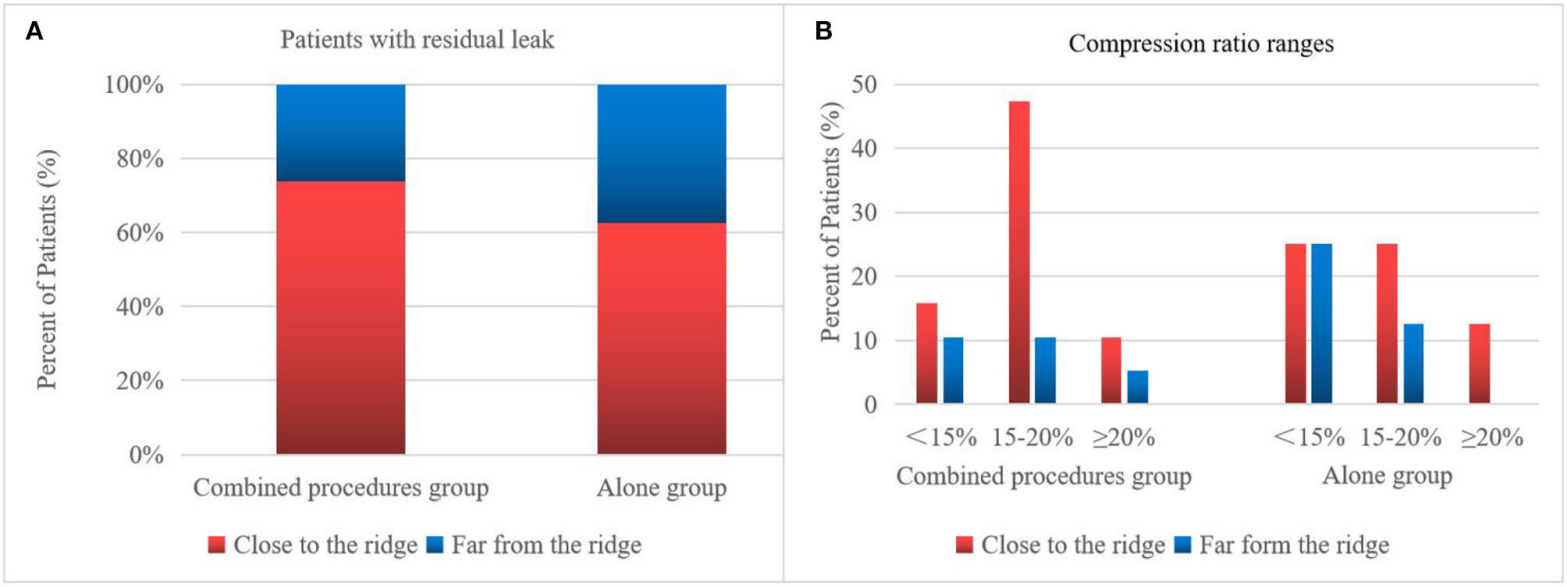
Residual leak of the WM device. The 6 week frequency and compression ratio are close to the ridge vs. far from the ridge. Patients close to the ridge in the combined procedures group have increased residual leaks and require a larger compression ratio. **(A)** Percentage of patients with a residual leak in the combined procedure group has more residual leaks close to the ridge than far from the ridge. **(B)** Degree of compression ratio on TEE immediately after WM implant of patients with residual leak 6 week follow-up in close to the ridge vs. far from the ridge.

On assessing the extent of the residual leak at a 6 week follow-up by TEE, 27 (27.6%) patients [19 (37.3%)] were identified in the combined procedure group and 8 (17.0%) in the alone group (*p* = 0.04), with some residual leak that could be measured in the complete cohort (Table 3). Residual leaks from the implants resolved in two patients in the combined procedure group and two patients in the alone group (*p* = 1.0). New residual leaks were observed in 13 patients in the combined procedures group and 4 patients in the alone group (*p* = 0.03). Finally, device compression ratios were not statistically different in the combined procedure and alone groups during the follow-up of TEE (Table 3), but in the combined procedures groups, between intra-procedural and 6 week follow-up, we observed a significant difference (22.44 ± 3.90 vs. 19.59 ± 5.39%; *p* = 0.03; Table 4). Device compression ratios were statistically different in the combined procedures group of residual leaks (22.75 ± 4.27 vs. 18.63 ± 5.12%; *p* = 0.02) between intra-procedural and 6 week follow-up (Figure 2). Device compression ratio was significantly different in the combined procedures group close to the ridge between intra-procedural and 6 week follow-up (22.63 ± 5.08 vs. 18.79 ± 4.92%; *p* = 0.02; Figure 3). A total of 14 patients (73.7%) with residual leak were close to the ridge in the combined procedure group and 12 patients (85.7%) of them had a compression ratio of < 20% (three patients < 15%, nine patients 15–20%) (Figure 4). Besides the combined procedures group that underwent CA, none of the characteristics (clinical, anatomic, or procedural) at 6 weeks were found as independent predictors of device leak.

**Table 4:** Device compression ratio at the time of implant and 6 weeks.

	**Device compression ratio at implant (%)**	**Device compression ratio at 6 weeks (%)**	**P-value**
Combined procedures group (*n* = 51)	22.44 ± 3.90	19.59 ± 5.39	0.03
Alone group	22.85 ± 3.72	21.58 ± 4.37	0.26

### 3.4. Pre-procedural complications

There were 3 (3%) patients (two in the combined procedures group and one in the alone group; *p* = 1.0) who had mild hematomas at sites of groin access and recovered without surgery. In 2 (2%) patients, tamponade or pericardial effusions without hemodynamic compromise were detected (one per group; *p* = 1.0). No atrioesophageal or arteriovenous fistula, embolization or intra-procedural device displacement, hemorrhagic/ischemic stroke, major bleeding events, or death was reported.

### 3.5. Follow-up results of clinical events

After LAAC, each of the 98 patients was followed up with at least one LAAC device implantation, TEE evaluation, and outpatient visit or telephonic clinical follow-up. The period of mean follow-up was equivalent (combined procedures: 25.2 ± 16.2 months vs. alone group: 22.4 ± 18.8 months; *p* = 0.15). All of the patients were given anticoagulants for 6 weeks before the first review of TEE 6 weeks after surgery, except for one patient with gastrointestinal bleeding who was given anticoagulation after 2 weeks of suspension. After the follow-up visit of 6 weeks, OACs were discontinued for 92 out of 98 (94%) of the implanted patients. Asymptomatic device thrombus was detected in one patient in the combined procedures group. In one patient (a 62-year-old man) with AF and LAA thrombus history, a 33-mm WM device was implanted immediately post-PVI ablation. After the combined procedure, rivaroxaban was taken for 1.5 months, and residual leaks (5.2 mm) and a thrombotic mass (11 × 6 mm) were detected. Rivaroxaban was continued, and 6 months after therapeutic OAC, the thrombus dissolved, although the residual leak was >5 mm. Thus, because of the unsuccessful LAA closure, the patient was continued on OAC for the study duration. There were no significant variations observed in systemic embolism, cardiovascular death, stroke/TIA, all-cause death, residual leak, or device thrombus (>5 mm) among the two groups (Table 3).

## 4. Discussion

### 4.1. Main findings

The major finding of our study is that, although combined procedures are safe, we found that the resolution of ridge edema caused by CA might cause an increased residual leak and a smaller device compression ratio. Therefore, for patients whose release site is closer to the ridge, a larger device should be selected to maintain the compression ratio.

### 4.2. Efficacy and safety of the combined procedure

As an effective alternative to OAC for stroke prevention, LAAC is now accepted in patients with AF at high thromboembolic risk and considerable bleeding (18). Swaans et al. demonstrated the safety and efficacy of combined procedures, where 30 patients with a high risk of stroke or bleeding and drug-refractory AF had successful CA and then LAAC in a single procedure. In the 1-year follow-up, there were no thromboembolic events (14). The experience was nearly similar in other studies conducted alone and that were multicentered (12, 13, 19). In our study, the combined procedure of CA along with LAAC was technically possible with no major complication risks. The combined procedure of CA and LAAC for patients AF who had a high stroke or bleeding risk appears to be a practical and advantageous option in comparison to the staged plan, with some procedures requiring catheterization of the transseptal and less risk of hospitalizations and procedure-related complications, shorter total required OAC duration, and promising decline in long-term stroke risk with no requirement for OAC (20). A combined procedure would enable improvements in compliance and quality of life for patients in comparison to the processes carried out in a staged fashion.

### 4.3. Combined procedure and residual leaks

Post-WM implant, residual leaks are identified during the follow-up. Residual leaks can potentially form intra-LAA thrombus followed by embolism stroke, particularly when the residual leak is set at >5 mm (21). The mechanisms for the presence of residual leaks are potentially device undersizing, non-circular LAA orifice shape, post-implant device migration, off-axis deployment, deficient endothelialization, or perpetual remodeling after LAA implant (21). Furthermore, the ultimate position of a deployed device may be dependent on the LAA morphology (22–24). However, in our series, the potential mechanisms mentioned above were not seen between the groups. In 2015, Alipour et al. showed a combined procedure in 62 patients, and by the end of the procedure, 12.9% of patients had minimal residual leaks, while 45% had minimal residual leaks after 60 days (25). Phillips et al. assessed the 30-day outcomes of the combined procedure by collecting data from two large registries of multicenter prospective LAAC (WASP and EWOLUTION). They analyzed 139 combined procedures; at the first TEE follow-up, new residual leaks were observed (2.9–39% from intra-procedure or post-procedure) (13). Wintgens et al. showed a minimal residual flow of < 5 mm in 7.4% of patients (*N* = 26), while 28.6% of implants were within 90 days post-procedure at the time of TEE (26). The new residual leaks at the follow-up TEE were observed to be higher for combined procedures than for a single LAAC procedure (27, 28). Du et al. found that the WM device was performed in 82 patients with symptomatic AF, with LAAC performed before (occlusion-first group, *N* = 52) or after (ablation-first group, *N* = 30) CA, and higher new residual leak rates were observed in the ablation-first strategy at follow-up (29). Hence, we considered that CA is associated independently with a fresh residual leak. In our study, significantly increased residual leaks were observed in the combined procedure patient group (< 5 mm) on a TEE follow-up of 6 weeks (37.3 vs. 17.0%). There were four possible reasons for higher residual leak rates: first, the formation of post-ablation edema may lead to an underestimated true LAA diameter and a consequently undersized WATCHMAN implant. Kita et al. previously reported that edema post-ablation may lead to haziness and difficulty in defining the border (30). Second, edema from ridge relieving leads to landing zone dilatation and progressive chamber impacting the device compression, which may continue after implant and contribute partly to the residual leaks observed on follow-up imaging. Our study further demonstrated significant differences in the compression ratio between intra-procedural and 6 week follow-up of combined procedure groups. Third, residual leaks might be partially masked by an edema response at the time of implant; however, after relief from edema, residual leaks may increase, or new residual leaks may appear. This phenomenon was also confirmed in our experiment, as shown in Figure 1. In these patients, only new residual leaks or increased residual leaks occurred, but the compression ratio was not significantly different compared with intra-procedural estimation. Fourth, AF affects LA remodeling after becoming sinus rhythm and the potential for a decrease or increase in any leak due to the remodeling of arteries in due course of time. In our experiment, the position of the WM device in 23 (45.1%) patients was far from the edematous region, and no remarkable difference was observed in the ratio of compression between intra-procedure and 6 week follow-up (Figure 3).

Our study confirms to the requirement of CA on the type specification selection of the device for the first time. A strategy of deliberate oversizing of the WM device by 20% or more rather than the initially recommended 10–20% may have contributed to the lower rates of residual leaks seen in recent registry studies (11, 31, 32), which was consistent with our study. While device stability could be facilitated by a high compression ratio, it may disturb the complete device expansion, which could cause imperfect LAAC. A suitable compression ratio was found to be 10–20% for patients with NVAF having WM device implantation, and the risk of residual leaks could increase with a lower or higher compression ratio (33). We observed that CA would increase the thickness of the ridge by 2–3 mm, so the device size should be increased by 2–3 mm from the original. In contrast, cryoablation is also a common treatment for paroxysmal AF and can also cause ridge edema. Ren et al. reported that distinct pulmonary vein ridge edema was observed after the cryoablation procedure by TEE (34). Considering that ablation can result in ridge edema, during TEE, if the edge of the device was found to be close to the ridge edema, the compression ratio should be as large as possible at ~20%.

### 4.4. Limitations

This was a retrospective, single-center study with a few limitations. First, while we showed an increased residual leak in the group of combined procedures, whether these patients had an increased risk of events is unclear; an adequately powered trial needs to be done. Second, several patients did not follow up in 1-year, so we could not draw meaningful conclusions on the rates of residual leaks between the follow-up period of the groups of over 6 weeks. Third, these data do not include whether LAAC affects the long-term outcome of CA in patients with AF.

## 5. Conclusion

This study indicates the feasibility of CA and LAAC combined treatment in a single procedure, which is safe and effective for patients with NVAF at high risk of chronic anticoagulation contraindications or stroke. Residual leak and smaller device compression may be partly associated with CA, which suggests that, if feasible, large sizing of the device may be preferred, especially close to the ridge.

## Data availability statement

The raw data supporting the conclusions of this article will be made available by the authors, without undue reservation.

## Ethics statement

The studies involving human participants were reviewed and approved by Yantai Yuhuangding Hospital. The patients/participants provided their written informed consent to participate in this study.

## Author contributions

XZ and HC designed the study. XZ, JL, HC, CW, and LZ performed the experiments. WL, LW, and LS perform the echocardiograph report. XZ and WL prepared the manuscript. All authors have seen and approved the final published version of this manuscript.
